# Effect of acupuncture on lung cancer-related fatigue: study protocol for a multi-center randomized controlled trial

**DOI:** 10.1186/s13063-019-3701-0

**Published:** 2019-11-09

**Authors:** Zhaoqin Wang, Shanshan Li, Luyi Wu, Qin Qi, Huirong Liu, Xiaoming Jin, Jianhui Tian, Ming Zhang, Xiaopeng Ma, Deli Sun, Shifen Xu, Huangan Wu

**Affiliations:** 1 Yueyang Hospital of Integrated Traditional Chinese and Western Medicine, Shanghai University of Traditional Chinese Medicine, Shanghai, 200437 China; 2 Shanghai Research Institute of Acupuncture and Meridian, Shanghai University of Traditional Chinese Medicine, Shanghai, 200030 China; 3 Shanghai Municipal Hospital of Traditional Chinese Medicine, Shanghai University of Traditional Chinese Medicine, Shanghai, 200071 China; 4 Stark Neurosciences Research Institute & Department of Anatomy and Cell Biology, Indiana University School of Medicine, Indianapolis, IN 46202 USA; 5 Longhua Hospital, Shanghai University of Traditional Chinese Medicine, Shanghai, 200032 China; 6 Shanghai Chest Hospital, Shanghai, 200030 China

**Keywords:** Acupuncture, Lung cancer, Cancer-related fatigue, RCT, Multi-center randomized controlled trial

## Abstract

**Background:**

Fatigue is one of the primary symptoms in lung cancer, with a prevalence of 88.0% in survivors of cancer, and an even higher prevalence post resection surgery. Effective fatigue control after lung cancer surgery is important for patient recovery and quality of life. Some studies have shown that acupuncture might be effective in treating cancer-related fatigue; however, randomized controlled trials (RCTs) of suitable sample size are limited.

**Method/design:**

This is a multi-center, patient-blinded RCT. A total of 320 eligible patients will be recruited in four centers and randomly assigned to either the acupuncture group or the sham acupuncture group in a 1:1 ratio. Treatment will be given twice per week for 12 sessions. Treatment will be given at acupoints GV20, GV29, CV12, CV6, CV4, and bilateral LI4, LR3, SP6, ST36. The primary outcome will be assessed using the Chinese version of The Brief Fatigue Inventory. The secondary outcomes will be measured using The European Organization for Research and The Treatment of Cancer Quality of Life Questionnaire, and the Hamilton Rating Scale for Depression. The primary outcome will be assessed at all main points (baseline, the 3rd week, the 6th week, and at follow up time points) and the secondary outcomes will be assessed at baseline and the 6th week. Intention-to-treat analysis will be used in this RCT.

**Discussion:**

This trial protocol provides an example of the clinical application acupuncture treatment in the management of lung cancer-related fatigue. If the acupuncture treatment protocol confirms that acupuncture is an effective and safe option for lung cancer-related fatigue, it can be adopted as a standardized treatment.

**Trial registration:**

Chinese Clinical Trial Registry, ChiCTR1900022831. Registered on 27 April 2019.

URL: http://www.chictr.org.cn/showproj.aspx?proj=37823

## Background

Cancer is a worldwide health epidemic with ever increasing morbidity and mortality rates. Partially driven by the modern lifestyle, cancer is currently the second leading cause of death worldwide [1–3]. Lung cancer ranks first in the incidence of malignant tumors in China; about 781,000 people suffer from lung cancer per year [4]. Cancer-related fatigue (CRF) is a common comorbidity of oncological disease, and seriously affects the quality of life and mental health of patients. In 2000, the National Comprehensive Cancer Network (NCCN) guidelines note [5] that CRF is characterized by a feeling of fatigue and lack of energy and includes physical, emotional, and cognitive fatigue. It is persistent and is not relieved through rest. It may be caused by cancer itself or cancer-related treatments, and is not proportional to activity [5, 6]. A study has shown that the prevalence of CRF is 88.0% in survivors of cancer [7]. The respiratory function of patients with lung cancer decreases significantly after lung cancer resection, which can lead to worsening fatigue. Treatment of symptoms may be helpful in lung cancer-related fatigue, such as regulating immunity, treatment of depression, nutritional support, improving sleep, and exercise. Yet there are few effective therapies available for treating CRF. Therefore, it is imperative to find an effective treatment that is without side effects.

Acupuncture therapy has been successfully used to treat many varied diseases for thousands of years. In fact, some research on acupuncture for lung CRF has revealed its advantages [8–10], include relieving symptoms, improving patient quality of life, and delaying the progression of cancer. However, most of these studies are of low value because of flawed methodology, such as inappropriate duration of treatment, sample size, and lack of blinding. The lack of objectivity makes the findings of those reports unreliable.

Faced with the limitations of current research, high-quality evidence is needed to confirm the effectiveness and safety of acupuncture for lung CRF. Therefore, through this proposed multi-center, randomized controlled trial (RCT), we aim to obtain evidence on the use of acupuncture in the treatment of lung CRF. The findings of this trial will provide useful information; this will be shared through publication and will provide an optimal acupuncture treatment protocol for lung CRF.

### Hypotheses

This trial aims to prove that acupuncture is an effective intervention that can relieve fatigue and improve the life quality of patients with lung cancer after lung cancer resection surgery.

### Objectives

The objectives of the study are:

1. To assess whether acupuncture is cost-effective in improving scores on the Brief Fatigue Inventory-Chinese (BFI-C) and European Organization for Research and Treatment of Cancer Quality of Life Questionnaire (EORTC QLQ-C30), when compared with sham acupuncture.
2. To compare the differences in improvement of mood, measured by the Hamilton Rating Scale for Depression (HAMD), between the intervention group and control group.
3. To determine the influence of the credibility of acupuncture on acupuncture treatment, as assessed by the Credibility of Treatment Rating Scale (CTRS).

## Methods/design

### Study design

**Fig. 1:**
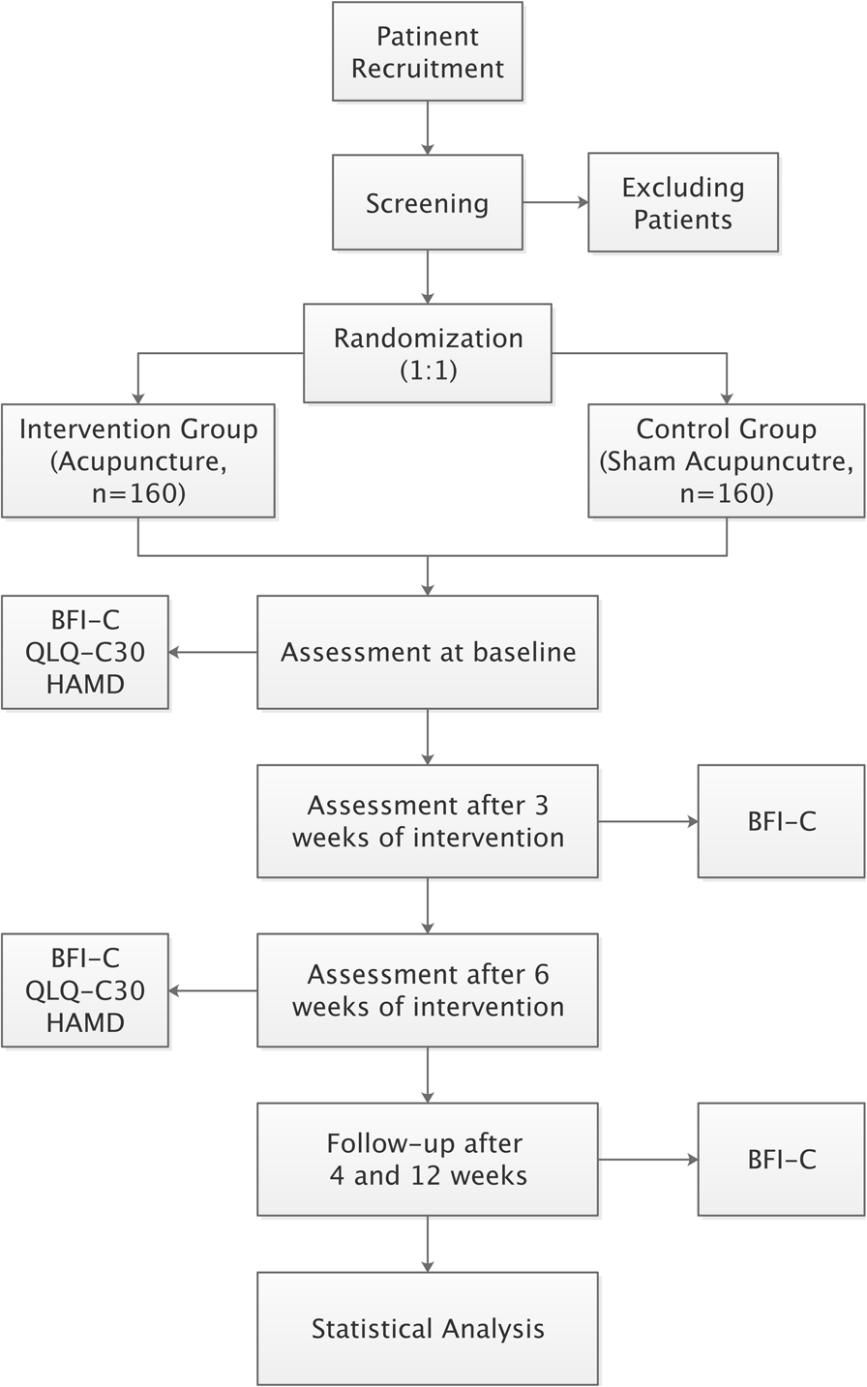
Flowchart of the study process. BFI-C, Brief Fatigue Inventory-Chinese; QLQ-C30, European Organization for Research and Treatment of Cancer Quality of Life Questionnaire; HAMD, Hamilton Rating Scale for Depression

We propose a multi-center, randomized, sham-controlled, patient and assessor blinded trial. The trial commence in four centers after ethical approval has been obtained from the Institutional Review Board. The clinical trial is designed and reported following the Consolidated Standards of Reporting Trials [11] and Standards for Reporting Interventions in Clinical Trials of Acupuncture guidelines [12]. Eligible patients will be randomly divided into the acupuncture group and the sham acupuncture group in a 1:1 allocation ratio. All participants will provide signed informed consent before proceeding with the trial. The flow chart of the study process is shown in Fig. 1.

### Sample size

The sample size calculation was based on change in BFI-C scores. According to previous studies [13], we assume the change is − 2.35 in the acupuncture group and − 2.0 in the sham-acupuncture group; therefore, the mean difference between two group is 0.35 with standard deviations of 1.03 and 1.00, and a sample size of 133 per group will provide 80% power to reject the null hypothesis with a significance level of 0.05, using a two-sided, two-sample, unequal-variance *t* test. Allowing for a dropout rate of 20%, a total of 320 participants should be recruited for this trial.

### Recruitment

This multi-center, randomized, sham-controlled, patient and assessor blinded trial will be conducted in the Shanghai Yueyang Hospital of Integrated Traditional Chinese and Western Medicine, Shanghai Municipal Hospital of Traditional Chinese Medicine, Shanghai Chest Hospital, and LongHua Hospital Shanghai University of Traditional Chinese Medicine. Participants of the study will be recruited through the outpatient clinic or inpatient, hospital-based Wechat advertising, and posters in the hospital. Any interested patients will be screened by telephone or on site and consented for the study. After being informed of the details of this RCT, participants will be asked to provide signed informed consent, and deliver it to an independent research assistant. Patients will be randomly divided into two groups: the acupuncture group (*n* = 160) and the sham acupuncture group (*n* = 160). All participants will undergo 6-week treatment with acupuncture (or sham acupuncture) and a series of assessments of CRF, free of charge. Timing of treatment assessments and data collection are as follows in Table 1.

**Table 1:** Timing of treatment assessments and data collection

	Study period					
	Enrollment	Baseline	Treatment phase		Follow-up phase	
Timepoint	-1 week	0 weeks	3 weeks	6 weeks	4 weeks	12 weeks
Enrollment						
Eligibility screen	X					
Informed consent	X					
Medical history	X					
Allocation		X				
Interventions						
Acupuncture		X	X	X		
Sham acupuncture		X	X	X		
Assessments						
Primary outcome						
BFI-C		X	X	X	X	X
Secondary outcomes						
EORTC QLQ-C30		X		X		
HAMD		X		X		
Others						
Adverse events		X	X	X		
Patients’ satisfaction				X		
Success of blinding				X		

*BFI-C* Brief Fatigue Inventory-Chinese, *EORTC QLQ-C30* European Organization for Research and Treatment of Cancer Quality of LifeQuestionnaire, *HAMD* Hamilton Rating Scale for Depression

#### Inclusion criteria

Participants with the following conditions will be included:

1. Participants in line with interpretation of Chinese lung cancer treatment guidelines (2015 edition) for the diagnosis of lung cancer [14]
2. Imaging evidence that supports a diagnosis of lung adenocarcinoma
3. Resection surgery performed within the last 3–6 months but no chemotherapy
4. Meet the diagnostic criteria for CRF [15] and BFI-C score > 4
5. Age 18–75 years
6. Karnofsky Performance Status (KPS) score > 80 [16]
7. Able to understand the nature of the study and willing to give informed consent
8. Capable of providing responses during outcome measurement

#### Exclusion criteria

Participants with the following conditions will be excluded:

1. Comorbidities such as serious heart, kidney, or liver disease
2. Severe mental disorder such as cognitive impairment
3. Other physiological or pathological causes of fatigue such as chronic fatigue syndrome (CFS)
4. Acupuncture received within the past 6 months
5. Pregnancy or currently lactating

### Randomization and allocation concealment

Stratified block randomization will be used in this experiment. An independent statistician will generate the randomization sequence using SPSS 23.0. The participants who meet the criteria will be randomly assigned to one of the two groups in 1:1 ratio by computer-generated random sequences after completion of the baseline assessment. Participants will be randomized in blocks of 4–8 at each center. The randomization will be concealed by using opaque envelopes. The treatment allocation codes will not be revealed until the participants have finished all baseline assessments and before the first acupuncture treatment. In order to minimize breaks in coding, the principal investigator (PI) who designed the trial and research personnel who perform the outcome assessments will be blinded to the treatment assignment. Only the acupuncturists will know which participants belong to which groups.

### Blinding

Participants will be informed that they will be randomly assigned to either acupuncture treatment or acupuncture-like simulation treatment. Participants will be asked to wear an eye-patch when they receive treatment: the two groups will receive identical treatment except no skin penetration will be involved in the sham acupuncture group. Therefore, participants and other researchers (the PI, data analysts, outcome assessors, and statistician) will be blinded to the group allocation. All researchers will be trained before the trial begins, to ensure the successful implementation of the blinding method.

### Intervention protocol

The acupuncture treatment will be conducted after all participants have given informed consent. All patients will receive 12 treatments, either acupuncture or sham acupuncture twice per week for 6 weeks. In each treatment session, every patient will be placed in a separate space and asked to wear an eye-patch. Each procedure will last 30 min and will be performed by a trained acupuncturist who will be a registered practitioner with more than 3 years of experience in clinical practice. The acupuncturist will confirm that the participant has undergone the assigned procedure. To improve patients’ compliance, all patients will receive the intervention and assessments by telephone reservation.

The acupuncture procedure will be performed following the Guidance of Clinical Practice of Acupuncture [17]. Before treatment, the patients’ skin will be sterilized using alcohol wipes, then the experienced acupuncturists will begin the treatment. In the treatment group, acupuncture needles will be standard stainless steel, sterile, and disposable (0.25 × 40 mm and 0.30 × 40 mm in length; Jia Jian, China). The Streitberger placebo-needle will be used at the same acupoints in the control group. The methods and acupoints for acupuncture treatment are shown in Table 2. Any concomitant care or intervention that has the effect of supplementing *Qi* (traditional Chinese medicine term) is not allowed to be used during the treatment and follow-up period, including using some special herbs and exercising Qi-Gong et al.

**Table 2:** Details of intervention

	Intervention group	Control group
Acupoints	GV20, GV29, CV12, CV6, CV4, LI4, LR3, SP6, ST36	GV20, GV29, CV12, CV6, CV4, LI4, LR3, SP6, ST36
Depth of insertion	GV20, GV29, LI4, LR3 10 mm	No insertion
	CV12, SP6, CV6, CV4, ST36 30 mm	
Needle type	Steel needle (Wuxi Jiajian Medical Co. Ltd. Wuxi, China)	Blunt-tip needle (Streitberger placebo-needle)
Needle sensation	With de-qi sensation	Without de-qi sensation
Electric stimulation	Needle on bilateral ST36 connected to SDZ-III Electronic Acupuncture Treatment Instrument (Hwato, China). with electric pulse at a frequency of 2.5 Hz and an intensity of 45 mA	Needle on bilateral ST36 connected to SDZ-III Electronic Acupuncture Treatment Instrument (Hwato, China). without electric pulse
Frequency and duration	Twice per week for 6 weeks	Twice per week for 6 weeks

### The acupuncture group

The acupuncture group will receive real acupuncture treatment for 6 weeks. According to our previous experience, *Baihui* (GV20), *Yintang* (GV29), *Zhongwan* (CV12), *Qihai* (CV6), *Guanyuan* (CV4), bilateral *Hegu* (LI4), bilateral *Taichong* (LR3), bilateral *Sanyinjiao* (SP6), and bilateral *Zusanli* (ST36) will be used as the acupoints for treatment. Patients will be treated in the supine position at GV20, CV12, CV6, CV4, and LI4 (with the needle tip pointing towards the ground), at CV29 and LR3 (with the needle tip pointing towards the feet), and at SP6 and ST36 (with the needle tip pointing towards the limb extremities). All the acupoints will be punctured perpendicularly to the respective depth of 10–30 mm, manipulating manually (including lifting, thrusting, and rotating) until the patient reports needling sensations (*Deqi* sensation). The needles on the bilateral *Zusanli* (ST36) will be connected to an SDZ-III Electronic Acupuncture Treatment Instrument (Hwato, China), using continuous wave type stimulation, with a frequency of 2 Hz, and intensity of 2~3 mA. Needles will be retained for 30 min.

### The control group

The control group will undergo treatment with special sham acupuncture. We will use a non-invasive placebo control, the Streitberger placebo-needle. Acupoints will be the same as in the acupuncture group but without insertion of the needle. Similarly, the electroacupuncture apparatus (SDZ-III Electronic Acupuncture Treatment Instrument) will be set beside the patients and connected to the bilateral *Zusanli* (ST36), without an electrical pulse. This set up will remain in place for the next 30 min.

### Outcome measures

We will assess the primary outcome at baseline, week 3, post treatment (week 6), and at follow up at the 4th and 12th weeks after the end of treatment. Secondary outcomes will be assessed at baseline and week 6.

### Primary outcome

#### Brief Fatigue Inventory-Chinese version (BFI-C)

The change in BFI-C scores will be used to evaluate the degree and impact of fatigue. The Brief Fatigue Inventory scale is designed by Mendoza [18] and translated by Wang [19], and it comprises two parts, (the first part consists of 1–3 items, the second part consists of 4–9 items), and questionnaire items 1–3 are used to assess the individual’s overall fatigue level over the past 24 h. The items 4–9 are designed to evaluate the effects of fatigue on general activities, emotions, walking ability, normal work (including work and housework), relationship with others, and enjoyment of life. The scale uses the 10-point scoring method, whereby 0 points indicates no fatigue and 10 points the most severe fatigue. A higher score indicates more severe fatigue. According to the average score, it can be divided into four grades, that is no fatigue (score 0), mild fatigue (score 1–3), moderate fatigue (score 4–6), and severe fatigue (score 7–10). The BFI scale has been validated in patients with different types of cancer all over the world [19–21]; the structural validity is 0.81–0.92, and the Cronbach’s coefficient of the total scale is 0.96. The Chinese version of the BFI has been proven to have good reliability and validity [19]. The mean of the change in score on the BFI-C at week 6 will be analyzed as the primary outcome.

### Secondary outcomes

#### European Organization for Research and Treatment of Cancer Quality of Life Questionnaire (EORTC QLQ-C30)

The EORTC QLQ-30 is a core scale developed by the European Organization for Research and Treatment (EO RTC) for the determination of quality of life in patients with cancer [22]. It includes a total of 30 entries, which are divided into 15 domains; among these there are 5 functional domains (body, role, cognition, mood, and social function), 3 symptom domains (fatigue, pain, and nausea and vomiting), 1 overall health status/quality of life area and 6 single entries (each as a domain). The higher the score for the functional domains and overall health status, the better the functional status and quality of life. The higher the score for the symptom domains, the worse the quality of life. The mean of any change in score at week 6 will be analyzed.

### Hamilton Rating Scale for Depression (HAMD)

The HAMD developed in 1960 [23, 24] is the questionnaire used to describe the severity of cognitive and bodily symptoms of depressive disorders. Each item is rated on 3-point or 5-point scales. According to the total score, depression can be divided into four grades: normal (total score < 7); patient may have depression (total score 7–17); depression (total score 17–24); and severe depression (total score > 24). The higher the total score, the more significant the tendency toward depression. The mean of any change in score at week 6 will be analyzed.

### Safety assessment

Any adverse events occurring during the trial will be recorded by the patients and physicians. Any discomfort, symptoms, or other diseases will be assessed. All details of adverse events will be reported in the case report form. The researcher will interview participants and write an adverse event report after treatment. The Data and Safety Monitoring Board and Ethic Review Board will assess any correlation between the adverse event and the intervention, and make the final decision as to whether to continue the study or not. We will analyze the influence of all events at the end of the trial.

### Credibility of Treatment Rating Scale (CTRS)

The CTRS is a scale for assessing the credibility of the acupuncture treatments. It consists of four items and is used to assess the participants as “perceived logic of the treatment,” “confidence in recommending the treatment to their friends who have similar complaints,” “confidence in the treatment to alleviate their complaint,” and “likelihood that the treatment would alleviate their other complaints.” A lower score indicates greater confidence in the received treatment [25, 26].

### Assessment of blinding 

After the final treatment session, the success of the blinding method will be tested by asking the participants the following question “When you volunteered for the study, you were informed that you had an equal chance of receiving traditional acupuncture or acupuncture-like simulation treatment. Our study is finished now, which style acupuncture do you think you are received?” The participants will be provided with three choices of response to this question: acupuncture, acupuncture-like simulation, or uncertain. If participants do not chose “uncertain”, we will ask the reason why they have made that assumption [27].

### Quality control

The Clinical Research Center of Drugs of Shanghai University of Traditional Chinese Medicine Data Monitoring Team will be responsible for controlling bias and identifying problems in the project. In order to guarantee the quality of this study, every acupuncturist in this trial will be a registered practitioner with 3–5 years of clinical experience in the practice of acupuncture. Meanwhile, a qualified clinical trial expert will monitor every trial center, and regular board meetings will be held to ensure the quality of the study process.

### Monitoring

According to recommendation of the National Institutes of Health (NIH), a Data and Safety Monitoring Board (DSMB) will be formed to monitor the trial progress and review the safety and quality of the data [28]. The committee consists of three members, including a senior acupuncturist, an oncologist, and a statistician (Additional file 2). The DSMB is independent of the proposed trial and all members will have to declare any conflict of interest in the trial. Regular meetings will be held during the trial, to ensure the data are collected scientifically and ethically. Moreover, the DSMB can ensure participants are not exposed to unnecessary risks, and has the right to unblind the study if serious adverse events related to the intervention occur. The DSMB will provide data monitoring with access to any interim results and will make the final decision to terminate the trial if necessary. It also will identify problems in the project, if any, make decisions on changing the details of this protocol, apply for approval from the Ethics Review Board by written application, and announce the persons conducting the trial by written notice after approval is received from the ethics committee. Besides this, a qualified clinical trial expert will be invited to monitor this study, and the PI will take full responsibility and make any final decisions.

### Clinical trial registration

This RCT was registered in the Chinese Clinical Trial Registry (ChiCTR1900022831) on 27 April 2019. The URL is http://www.chictr.org.cn/showproj.aspx?proj=37823.

### Data management

The participant will be interviewed at each time point, and further cancer care advice will be given at the interview. This procedure will promote retention of participants and completion of follow-up assessments. All the original data will be collected by blinded assessors and double-entered into the electronic data capture system (EDC). The system will be tested before it is officially launched to ensure that it meets the trial requirements. The original data recorded in case report forms will be entered into the EDC within 1 week after the participants have finished all the treatments and follow-up assessments. Codes and initials will be used in the case report forms, instead of the participant’s information, to protect the participant’s privacy. If the data are found to be uncertain, the data supervisor will notify the researcher to respond with a data question form. If necessary, the statistician will send a data question form to the researcher and the researcher’s answer will be entered on the form. The inspector will then return the question form to the statistician.

### Statistical analysis

Statistical analysis of data will be carried out using SPSS 23.0 software. We will use multiple imputation to address any missing data. An independent statistician, who will be blinded to group allocation, will analyze all data, including those from any participants who drop out during the trial, using the intention-to-treat (ITT) method. Baseline demographics will be analyzed descriptively. Changes in BFI-C will be compared between the two groups as the primary analysis, using repeated measures analysis of variance (ANOVA (general linear model)). For the secondary outcomes, scores on the QLQ-C30, HAMD, and CTRS will be compared between the two groups using Student’s *t* test or the Wilcoxon rank-sum test. All reported *P* values will be two-sided, and a *P* value of less than 0.05 will be considered statistically significant. The mean difference and confidence intervals at the 95% level will also be calculated using SPSS.

## Discussion

Fatigue is a typical symptom that is common in lung cancer and can seriously affect a patient’s quality of life and emotional health [29, 30]. CRF is a common condition that occurs at different stages of oncological disease (after surgery, treatment with target therapy, etc.) [29–31]. With the development of complementary and alternative medicine, more and more patients with cancer tend to choose acupuncture as a main treatment to treat fatigue. Recently, several studies have shown that acupuncture can effectively improve the symptoms of CRF [32–36]. However, there is currently a lack of multi-center, large-sample, sham-controlled, RCTs in CRF [29, 37]. In this study protocol, we designed a randomized, multi-center, sham-controlled, patient and assessor blinded trial (Additional file 1). If the protocol confirms that acupuncture is effective and safe, it can be implemented for relieving CRF and improving quality of life in patients with lung cancer.

Because of the nature of acupuncture, there are many factors that can affect the clinical outcomes, including individual differences, needle insertion techniques and duration of the sessions. Our study protocol is based on literature from textbooks and relevant reports, and on our previous clinical experience. Traditional acupoints will be selected for treatment (GV20, GV29, CV12, CV6, CV4, and bilateral LI4, LR3, SP6, and ST36). Another advantage is that the same trained acupuncturists will implement a standardized protocol for point selection and for the needle insertion, minimizing factors that might affect the clinical outcomes. The nature of acupuncture treatment made double-blinding impossible, but our design incorporating sham acupuncture allows blinding of the patient and assessor. Only the acupuncturist will know the treatment allocation, but they will know nothing about the results, and assessors do not know the treatment allocation before and after the evaluation of treatment efficacy, thus avoiding the potential for bias. Another innovation in this study protocol is the use of the CTRS to assess the influence of the patients’ perceptions of the credibility of acupuncture on acupuncture treatment.

However, this study protocol still faces several limitations and challenges. The first is the blinding method. Because of the nature of the clinical trials of acupuncture, it is inevitable that the acupuncturist will know the treatment allocation. However, the acupuncturists will be blinded to the results. In order to prevent the acupuncturist from accidentally revealing the group allocation, their interactions with the patients will be limited. All patients will be asked to wear an eye-patch and arranged in a separate space during treatments. Overall, both groups will receive blinding to balance the efficacy between the two groups. The second limitation is the application of the acupuncture method. Before the study begins, all acupuncturists will receive several training sessions on the standardized protocol for point selection and needle insertion to ensure the consistency of the acupuncture techniques. The third limitation is the challenge of compliance. To solve this problem, we will provide patients outreach over the Wechat or phone to arrange the treatment time to improve patients’ attendance. Following completion of the trial, researchers will conduct follow up by phone to collect data on patients’ final outcomes.

Though there are many challenges, we will strive to standardize the steps of the trial and the quality of the trial will be monitored by the DSMB. We hope that this trial will provide a strong quality of evidence on the efficacy and the safety of acupuncture for treating lung cancer-related fatigue (CRF). We expect that our findings will advance knowledge in China on the effectiveness of acupuncture in patients with lung cancer who have CRF.

### Trial status

The first investigators’ meeting took place on 18 November 2018. The protocol version 2.0, which was revised on 15 April 2019, was approved by the Ethics Committee of Yueyang Hospital of Integrated Traditional Chinese and Western Medicine. The RCT is in preparation now and will launch on 1 June 2019. Recruitment is expected to end late 2021.

## Supplementary information

**Additional file 1.** SPIRIT 2013 Checklist: Recommended items to address in a clinical trial protocol and related documents*.

**Additional file 2.** Data and Safety Monitoring Board members.

## Data Availability

The trial results will be published through publication in a peer-reviewed scientific paper and poster or oral presentations in conferences. All data will be available from the 3rd month to the 3rd year after publication of the results with researchers who provide a methodologically sound proposal to achieve aims in the approved proposal. The trial data will be available from the corresponding author upon reasonable request.

## Abbreviations

ANOVA: Analysis of variance
BFI-C: Brief Fatigue Inventory-Chinese
CFS: Chronic fatigue syndrome
CN: Conception vessel
CRF: Cancer-related fatigue
CTRS: Credibility of Treatment Rating Scale
DSMB: Data and Safety Monitoring Board
EDC: Electronic data capture system
EO RTC: European Organization for Research and Treatment
EORTC QLQ-C30: European Organization for Research and Treatment of Cancer Quality of Life Questionnaire
GV: Governor vessel
HAMD: Hamilton Rating Scale for Depression
ITT: Intention to treat
KPS: Karnofsky Performance Status
LI: Yangming large intestine channel of hand
LR: Liver channel of foot Jueyin
NCCN: National Comprehensive Cancer Network
NIH: National Institutes of Health
PI: Principal investigator
RCT: Randomized controlled trial
SP: Spleen meridian of foot Taiyin
ST: Stomach channel of foot-Yangming
