# Supplementary material for: Effect of acupuncture on lung cancer-related fatigue: study protocol for a multi-center randomized controlled trial
Source: Trials. 2019 Nov 9;20:625. doi: 10.1186/s13063-019-3701-0 (PMC6842537; doi:10.1186/s13063-019-3701-0)
Supplement: Supplementary file 2 — Additional file 2. Data and Safety Monitoring Board members. [file 13063_2019_3701_MOESM2_ESM.docx]

**Data and Safety Monitoring Board members**

Lixing Lao, Ph.D, Professor, Acupuncturist

School of Chinese Medicine, The University of Hong Kong, Hong Kong, China

E-mail: [lxlao1@hku.hk](mailto:lxlao1@hku.hk)

Peng Zhang, Ph.D, Associate Professor, Thoracic Surgeon

Shanghai Pulmonary Hospital, Tongji University, Shanghai, China

E-mail: zhangpeng1121@tongji.edu.cm

Wei Zhang, Ph.D, statistician

Departement of Biostatistics, School of Public Health, Fudan University, Shanghai, China

E-mail: weizhang@shmu.edu.cn
